# Abstract of the Proceedings of the Esculapian Society, at the Meeting Held in Paris, Ill., January 1st and 2d, 1861

**Published:** 1861-01

**Authors:** D. W. Stormont

**Affiliations:** Secretary


### PROCEEDINGS OF SOCIETIES.

*Abstract of the Proceedings of the Esculapian Society, at the Meeting held in Paris, Ill., January* 1st *and* 2d, 1861.

(Reported by D. W. Stormont, M. D., Secretary.)

The Society met at the office of Dr. Davis, in Paris, Jan. 1st, and was called to order by the President, Dr. Chambers, at 10 o'clock A. M. The minutes of the last meeting were read and approved.

Dr. Geo. Ringland was elected a member of the Society.

Dr. Herrick, the Treasurer, submitted his report, which was received.

Dr. Stormont read a paper on the use of Anhydrous Sul. Zinc, as a Caustic in the treatment of malignant and semi-malignant ulcers—claiming for it escharotic properties, equal to arsenious acid or Chloride of Zinc, but free from the objections which apply to these; it being perfectly safe, easily managed, and its application attended with far less pain than any other powerful escharotic known.

Dr. York, Chairman of the Committee on Indigenous Botany, read a report on "The Hop," as an anodyne, tonic, and antiperiodic; possessing great virtues in the latter stage of Phthisis, in Spinal Irritation, in Stomatitis Materna, in Intermittant Fever, especially its chronic form, and in many cases of Gastralgia; is a most valuable antihectic; hence, useful in all diseases attended with hectic fever.

Dr. Chambers read a paper on Diphtheria, the main points of which were, that it is a blood disease, produced by a specific poison which manifests itself locally in the throat, as the virus of Small-Pox does upon the skin; with a tendency to spread upon the mucous surfaces. It is a disease of debility and is not contagious. Treatment, internally, Chlorate Potash and large doses of Sulph. Quinine. As a local application, Nitrate Silver, gr. 60, to one ounce of water. If the air passages are involved, the atmosphere in the room should be kept saturated with the vapor of boiling water.

Dr. Davis coincided generally with the essayist; preferred tinct. iodine for local application; believed it would stop the spread of the disease from the throat to contiguous parts.

Dr. Stormont had seen but little of this disease; believed it to be a blood disease, asthenic in its tendencies, demanding a supporting treatment. Some experiments he had read, favored the opinion that the specific virus, acting upon and through the blood, tends to induce an abnormal alkalinity of all the secretions, indicating the use of acid tonics, such as nitro-muriatic acid, or sulphuric acid, or *muriated tincture of iron*, with Quinine, nourishment and stimulants.

Dr. York said the danger lay not in the diseased condition of the blood, but from the extension of the disease downward into the windpipe, producing croupy symptoms. He relied upon tinct. iodine to stop this extention. It is not contagious. Treat according to the indications present. Some cases are sthenic, and others are asthenic. Or the same case may be sthenic to-day and asthenic to-morrow. Hence in one stage murcurials and sedatives may be demanded; in another stage tonics and stimulants are the remedies. Emetics should be used cautiously; of these he prefers alum, one teaspoonful every twenty minutes until vomiting. Avoid the too frequent application of caustics to the throat; it produces mischievous irritation; not oftener than once every day, or every other day. When the disease has extended into larynx and trachia, stop the local application of tinct. iodine, or Nit. Silver. It is then a useless annoyance. A vast majority of cases die, in which there is complete aphonia; known of only four recoveries from this condition. Here I give Quinine very freely, with Dover's powders.

Dr. Tenbrook. This disease is asthenic in character. Treatment; First order a mild aperient, then follow with stimulants and tonics. As a local application to the throat, greatly prefer the solid Nit. Silver; if used in. time it will prevent the extention of the disease into the windpipe; it should be used not oftener than once in twenty-four or forty-eight hours. In the interval use a gargle of common salt, sweetened. In the worst cases I have met with, aphonia and general venous congestion were present, when first seen, without much or any disease in the throat. Such generally die. Always have the patient washed all over daily with warm salt and water, and rubbed with a dry towel until the skin glows. Am not decided as to its being contagious.

Dr. Herrick reported a case of compound fracture of the leg.

The Business Committee submitted a report, which, after amendment, was adopted as follows, viz:

*Resolved*, That a subject shall be settled at each meeting, for general discussion at the subsequent meeting.

*Resolved*, That the question at the next meeting shall be; Is the character of diseases at this time asthenic?

Dr. Chambers was appointed to open the discussion.

The following standing committees were appointed, for the present year, viz:

ON PRACTICAL MEDICINE.

Dr. L. L. Todd.
" D. W. Stormont.
" D. O. McCord.

ON MIDWIFERY.

Dr. H. R. Payne.
" C. Johnson.
" J. M. Steele.

ON SURGERY.

Dr. O. Q. Herrick.
" H. W. Davis.
" W. M. Chambers.

ON EPIDEMICS.

Dr. J. Van Dyke.
" C. Duncan.
" C. Gorham.

ON INDEGINOUS BOTANY.

Dr. S. York.
" J. Tenbrook.
" F. R. Payne.

The following were the officers elected for the ensuing year, viz:

Dr. Tenbrook, President.
" J. Van Dyke, Vice Pres.
" D. W. Stormont, Secretary.
" J. M. Steele, Treasurer.

Drs. York, Chambers, Todd, H. R. Payne and Pearman, Censors.

Dr. Van Dyke was appointed to deliver the next public address.

On motion, the Society adjourned to meet in Charleston, on the last Wednesday in May.

